# Granulomatosis with Polyangiitis Localized in the Greater Omentum

**DOI:** 10.1155/2018/6145903

**Published:** 2018-02-28

**Authors:** Ryuji Komine, Takashi Kobayashi, Hiro Uehara, Keisuke Minamimura, Kazuhiko Mori, Toru Hirata, Atsushi Shimizu, Masaya Mori

**Affiliations:** ^1^Department of Gastroenterological Surgery, Mitsui Memorial Hospital, Tokyo, Japan; ^2^Department of Pathology, Mitsui Memorial Hospital, Tokyo, Japan

## Abstract

Granulomatosis with polyangiitis (GPA) is known as anti-neutrophil cytoplasmic antibody- (ANCA-) associated small vessel vasculitis and typically manifests as pulmonary-renal syndrome, but the disease is not limited to pulmonary or renal systems. The inflammation can involve whole body organs. In addition, the ANCA titer does not always become positive. Here, we describe the case of a 91-year-old man who presented with umbilical pain and fever of unknown origin. Only the increased computed tomography value of the greater omentum suggested intra-abdominal inflammation; however, serological examinations, including the ANCA level, could not reveal the focus or cause of symptoms. Finally, the histopathological examination of specimens surgically excised from the greater omentum demonstrated GPA limited to the greater omentum. This report reminds physicians to consider GPA in the differential diagnosis of acute abdominal pain or fever of unknown origin.

## 1. Introduction

Granulomatosis with polyangiitis (GPA) is a rare autoimmune disease, previously known as Wegener's granulomatosis, characterized by a pauci-immune necrotizing vasculitis of small- and medium-sized vessels that in typical cases involve the upper and lower respiratory tracts and the kidneys [1], although other limited involvements can be seen in some patients [2, 3]. GPA may occur at all ages, especially in the sixth and seventh decades of life. The classic triad of symptoms affects the upper respiratory tract, lungs, and kidneys, but the disease can affect any organ. Although in 80–95% of the patients the first symptoms are otorhinolaryngological manifestations of neck and head [4], patients may present with constitutional symptoms, including but not limited to fatigue, anorexia, and weight loss. These symptoms last for weeks to months with no evidence of specific organ involvement. On average, patients will be diagnosed within 3–12 months from the onset of symptoms, and at the time of diagnosis, more than two organ systems are involved [5].

The serum titer of the anti-neutrophil cytoplasmic antibody (ANCA) is known to be increased in most patients with GPA, and it is used as a biomarker. However, several patients have not demonstrated an increase in the serum ANCA titer [6]. In this report, we described a case of ANCA-negative GPA that was limited to the greater omentum. Although rare, necrotizing vasculitis should be considered in the differential diagnosis of acute abdominal pain or fever of unknown origin.

## 2. Case Report

A 91-year-old man presented with abdominal pain and was followed up to 2 days. On physical examination, periumbilical tenderness was found, but there were no peritoneal irritation signs or other obvious abnormal findings of the ears, nose, or skin. The blood test results showed a high inflammatory reaction (white blood cell (WBC) count 13,800/*µ*L; C-reactive protein (CRP) level 33.5 mg/L) and acute kidney injury (creatinine (Cre) 1.11 mg/dL). Urinalysis was normal. The chest radiography demonstrated no remarkable change. The computed tomography (CT) scan was negative for any infectious focus or other abnormal findings, such as cavitating nodules in the lungs or other site neoplasms. Antibiotics (tazobactam/piperacillin 13.5 g/day) were administered as empiric therapy.

**Figure 1:**
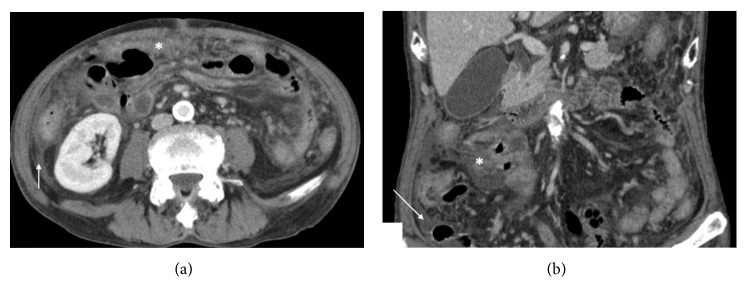
Abdominal computed tomography (CT) scan taken again after the patient showed the peritoneal irritation sign, revealing an increased CT value of the greater omentum (^∗^) and ascites (→). However, no infectious focus or neoplasms are observed.

After hospitalisation, the patient developed fever over 39°C. Results of the blood cultures and other cultivation tests were all negative, including the tuberculosis test. Additional analyses demonstrated a negative ANCA titer, proteinase 3- (PR3-) ANCA, and myeloperoxidase- (MPO-) ANCA. Two days later, the patient showed a new peritoneal irritation sign at the periumbilical and right lower abdomen. The CT scan showed an increased CT value of the greater omentum and ileocolic mesentery, and ascites (Figure 1). Blood test results showed that the inflammatory reaction was still high (WBC count 20,700/*µ*L; CRP level 29.3 mg/L), although renal function returned to normal (Cre level 0.93 mg/dL). The antibiotics were changed (meropenem 3 g/day), but the fever and abdominal pain did not disappear, and inflammatory reaction remained at a high level.

**Figure 2:**
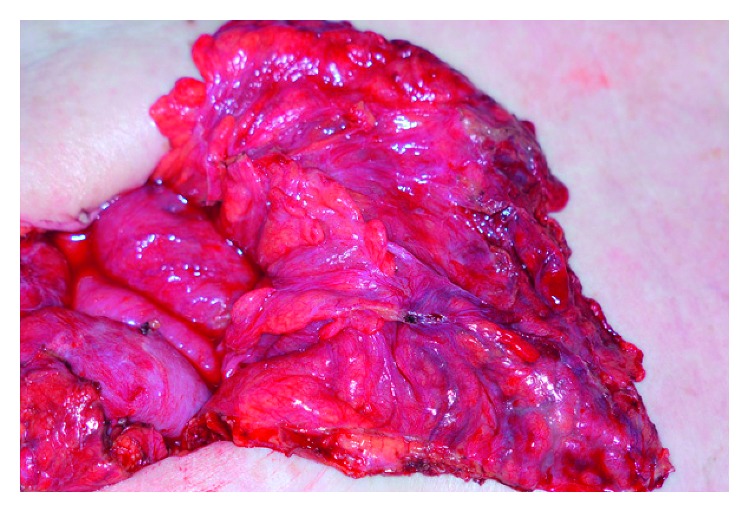
Diffuse redness and swelling of the greater omentum is shown, and ascites are present with minimal blood dominantly in the Douglas fossa and right paracolic gutter.

**Figure 3:**
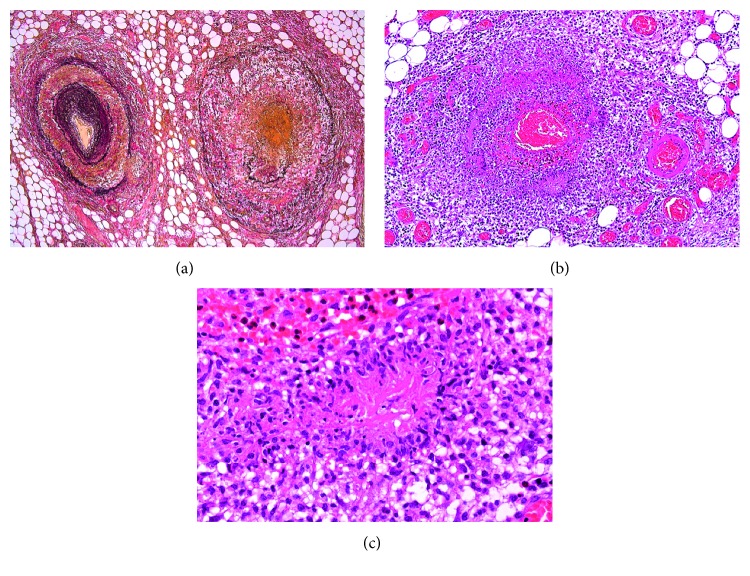
Mononuclear cell and neutrophil infiltration to the perivascular and vascular walls. (a) Vessel wall destruction is more severe in the vein than in the artery. (b) The vessels are surrounded by epithelioid granuloma and multinucleated giant cells. (c) Palisading granuloma is seen.

Because there was no sign of infectious focus for fever and abdominal pain, except for an increased CT value of the omentum and ascites, examination laparotomy was planned. Laparotomy revealed diffuse redness and swelling of the greater omentum and pale haemorrhagic ascites (Figure 2). Histological examination of the resected omentum demonstrated both arteritis and more severe venulitis, focal necrotizing vasculitis of the venules, and palisading granuloma (Figures 3(a) and 3(c)).

The patient was diagnosed as having GPA based on the Chapel Hill Consensus Conference 2012 definitions [7] and treated with intravenous methylprednisolone pulse therapy (1000 mg/day for 3 days). Subsequently, daily prednisolone was administered, and the dose was gradually tapered to 15 mg over 1 month. The patient was discharged without relapse.

One year later, the patient did not show any symptoms, and the follow-up CT scan showed no sign of vascular inflammation.

## 3. Discussion

Although GPA typically involves the upper airways, lungs, and kidneys, inflammatory destructive lesions may develop in almost all organs [1]. Severe gastrointestinal involvement has been reported occasionally [2, 3], but most of the cases were detected by necropsy. Walton reviewed 56 autopsy cases and found focal necrotizing arteriolitis in the intestine in 24% of the cases [8]. In most of the cases, GPA starts with nonspecific symptoms, such as fatigue, joint pains, and sinusitis. Occasionally, protracted fever without localising signs is manifested, but this is uncommon. In fact, pyrexia of unknown origin is less common in collagen vascular disease than in infectious disease. In a retrospective cohort of 857 patients, 16% had fever due to collagen vascular disease, and only 0.3% had fever secondary to GPA [9].

GPA is one of several vasculitides that are associated with ANCA, but it is not always associated with a positive ANCA titer. The ANCA titer becomes positive in approximately 82–94% of patients with GPA or microscopic polyangiitis patients according to the severity of the disease [10, 11]. GPA is usually associated with PR3-ANCA, and the specificity of ANCA testing can be as high as 90% in the active phase of generalised GPA [12]. Currently, the clinical diagnosis is usually based on the presence of distinctive ANCA and biopsy of the affected organ, but in the limited type of GPA, the ANCA titer occasionally becomes negative [13]. Positive levels of PR3-ANCA are found in only 46–70% of patients with the nonsystemic type of GPA [14]. The limited type of GPA has been reported to be associated with Th1 lymphocyte polarisation, as opposed to the generalised form with greater Th2 lymphocyte polarisation [15]. A biopsy is necessary to avoid overlooking ANCA-negative GPA, but over 50% of specimens may not be sufficient for making a diagnosis; thus, in some patients, repeated biopsies are necessary.

In our case, the patient presented with an atypical course of GPA. Except for fever and localized abdominal pain, the patient showed no issues of the respiratory tract or kidney. PR3-ANCA and MPO-ANCA titers were both negative. If the CT value would have shown no relevant change, it might have been difficult to plan examination laparotomy. During laparotomy, the omentum was obviously involved, and its histological examination showed necrotizing vasculitis of the venules and palisading granuloma. Because of these findings, the patient was diagnosed as having GPA classified as other single organ vasculitis according to the Chapel Hill Consensus Conference 2012 definitions [7].

Some reports assessed the clinical value of ^18^fluorodeoxyglucose-positron emission tomography (FDG-PET)/CT imaging to initially diagnose GPA by indicating the biopsy sites [16, 17]. However, almost no literatures have mentioned how to determine the biopsy site in the limited type of GPA because most cases had positive ANCA or typical symptoms and laboratory changes in the respiratory tract or kidney. FDG-PET/CT and other investigative methods to help determine the appropriate biopsy site should be further addressed.

ANCA-negative GPA shows various responses to the medical treatment. Holle analysed 50 localized GPA patients with prospective follow-up and reported that although the rate of complete remission was 34%, the relapse rate was 46% [14]. Among various drugs used for ANCA-negative GPA, prednisolone and cyclophosphamide are effective in some cases. As for our case, the sole administration of methylprednisolone was effective. Recently, rituximab has been reported to be able to control other cases in which these agents are ineffective [18, 19].

Although rare, necrotizing vasculitis should be considered in the differential diagnosis of acute abdominal pain or fever of unknown origin because, like in our case, an early diagnosis could contribute to not only treatment but also the prevention of severe abdominal manifestations, such as perforation or intra-abdominal bleeding.
